# BU-DLNet: Breast Ultrasonography-Based Cancer Detection Using Deep-Learning Network Selection and Feature Optimization

**DOI:** 10.3390/bioengineering10070825

**Published:** 2023-07-11

**Authors:** Amad Zafar, Jawad Tanveer, Muhammad Umair Ali, Seung Won Lee

**Affiliations:** 1 Department of Intelligent Mechatronics Engineering, Sejong University, Seoul 05006, Republic of Korea; amad@sejong.ac.kr; 2 Department of Computer Science and Engineering, Sejong University, Seoul 05006, Republic of Korea; jawadtanveer@sejong.ac.kr; 3 Department of Precision Medicine, School of Medicine, Sungkyunkwan University, Suwon 16419, Republic of Korea

**Keywords:** breast ultrasonography (BU), optimization, wrapper-based method, breast cancer (BC), image processing

## Abstract

Early detection of breast lesions and distinguishing between malignant and benign lesions are critical for breast cancer (BC) prognosis. Breast ultrasonography (BU) is an important radiological imaging modality for the diagnosis of BC. This study proposes a BU image-based framework for the diagnosis of BC in women. Various pre-trained networks are used to extract the deep features of the BU images. Ten wrapper-based optimization algorithms, including the marine predator algorithm, generalized normal distribution optimization, slime mold algorithm, equilibrium optimizer (EO), manta-ray foraging optimization, atom search optimization, Harris hawks optimization, Henry gas solubility optimization, path finder algorithm, and poor and rich optimization, were employed to compute the optimal subset of deep features using a support vector machine classifier. Furthermore, a network selection algorithm was employed to determine the best pre-trained network. An online BU dataset was used to test the proposed framework. After comprehensive testing and analysis, it was found that the EO algorithm produced the highest classification rate for each pre-trained model. It produced the highest classification accuracy of 96.79%, and it was trained using only a deep feature vector with a size of 562 in the ResNet-50 model. Similarly, the Inception-ResNet-v2 had the second highest classification accuracy of 96.15% using the EO algorithm. Moreover, the results of the proposed framework are compared with those in the literature.

## 1. Introduction

Breast cancer (BC) is the most prevalent malignancy among women worldwide. According to a 2020 statistics report published in 2021 [1], approximately 2.3 million new cases of BC are reported, accounting for 11.7% of the total number of cancer cases. With 685,000 fatalities, it is the fifth most common cause of cancer-related mortality worldwide. A good screening program can identify BC early, lower the risk of local and long-term recurrence, and enhance the five-year survival rate [2]. In accordance with national and international standards, women aged 40–74 years should receive a mammogram every year [3]. However, for those with dense breasts, the false-positive and false-negative rates are relatively high, which increases the likelihood of missed diagnoses. Breast ultrasonography (BU) is a complementary diagnostic technique not constrained by the type of glandular breast tissue [4]. It is particularly suitable for Asian women with dense breasts, as it boosts the detection rate of BC by 17% and lowers the chances of needless biopsies by 40% [5]. In addition to mammography, clinical examinations, and needle biopsies, BU is crucial for the assessment of breast diseases. Compared with other methods, BU has the advantages of being noninvasive, nonradioactive, and cost-effective [6]. However, the sonographer’s experience significantly affects BU diagnosis [7,8]. Complex structures, including normal tissues, cysts, benign lesions, and malignant tumors, are frequently observed in BC images. It can be difficult to distinguish between these various structures and precisely detect probable cancers; this requires expert knowledge and training.

Artificial intelligence (AI) and computer-aided methods have grown rapidly in recent years. Individualized analyses using AI can help doctors make better clinical decisions [9,10,11,12,13]. Deep learning is a subfield of AI that enables high-throughput correlations between images and clinical data by automatically identifying image patterns [14,15,16,17]. Therefore, efficient computer-assisted techniques are necessary for the highly accurate automated identification of BC [18,19].

Rezaei [20] recently reviewed various automatic and semiautomatic BC detection, segmentation, and classification approaches. Kwon et al. [21] analyzed the effectiveness of two- and three-view scan approaches. They concluded that the two-view scan approach outperformed the three-view scan approach. In another study [22], deep-learning networks were designed to distinguish between benign and malignant BU images. The developed model had a 95% area under the curve. Zhuang et al. [23] proposed an image fusion method to train pre-trained models to differentiate between benign and malignant BU images. They reported a high classification rate of 95.48%. Similarly, Zhang et al. [24] utilized pre-trained deep-learning models to detect malignancies. The parameters were fine-tuned using an optimization algorithm, resulting in a classification accuracy of 92.86%. Various pre-trained networks have also been utilized for the classification of BU images, resulting in high accuracy rates [25]. However, the primary drawback of these models is their long training time. In addition, the authors designed models for only two classes of problems (benign and malignant). Nevertheless, the model must be capable of distinguishing between the three classes of BU images (benign, malignant, and normal). To address this issue, authors of one study designed a 3D deep-learning model and achieved a high classification rate of 97.7%, albeit with a prolonged training time [26]. Moreover, conventional classifiers were trained to use the morphological features of the BU images to address the training time issue [27]. In a recent study [28], deep features were computed using the ResNeT-101 structure to train a support vector machine (SVM) model. The area under the curve was used as the evaluation metric. Nevertheless, the model could only categorize the BU images into two subclasses. In another study [29], the filter-based ReliefF algorithm was used to remove redundant deep features extracted from DenseNet-201 and MobileNet-v2 to train an SVM model (for three-class problems). The authors reported an accuracy of 94.57% and 90.39% for the augmented and original datasets, respectively. However, the features used in the filtering approaches were selected based on their relevance to the dependent variables. In certain circumstances, a threshold value must be selected to exclude extraneous information because it cannot link the features to the model performance. Therefore, further research is required to design a resilient and highly accurate model with short training times to classify BU images.

This study proposes a BU image-based framework for the diagnosis of BC in women. The highlights of the proposed methodology are as follows.

- Various pre-trained deep-learning models were utilized to compute the deep features of the BU images.
- Ten wrapper-based optimization algorithms were employed to compute the optimal subset of deep features, as follows: the marine predator algorithm (MPA), generalized normal distribution optimization (GNDO), slime mould algorithm (SMA), equilibrium optimizer (EO), manta-ray foraging optimization (MRFO), atom search optimization (ASO), Harris hawks optimization (HHO), Henry gas solubility optimization (HGSO), pathfinder algorithm (PFA), and poor and rich optimization (PRO).
- An SVM-based cost function was used to classify the BU images into subclasses (benign, malignant, and normal).
- Furthermore, a network selection algorithm was employed to determine the best deep-learning network.
- An online BU dataset was used to test the proposed methodology. Moreover, the findings of the proposed methodology are compared with those in the literature.

## 2. Methods and Materials

### 2.1. Breast Ultrasonography (BU) Dataset

This study used an online collection of BU images as the dataset [30]. The collection comprised 780 BU images from 600 women aged 25–75 years. Table 1 presents further information regarding the dataset.

### 2.2. Deep Feature Extraction Using a Pre-Trained Convolutional Neural Network

**Figure 1:**
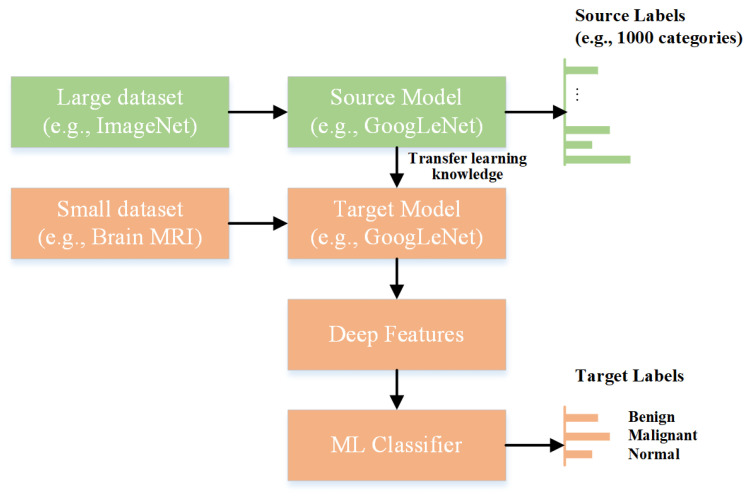
Extraction of deep features using a pre-trained deep-learning model.

The term “features” refers to the various characteristics that distinguish between different image classes. Choosing the essential attributes with the most significant fluctuations between the images can considerably improve the classification accuracy. The extraction of useful characteristics from images is a key operation that can be performed manually or with the help of a convolutional neural network. The accuracy of the manual feature extraction approach depends on image diversity, and it also takes a considerable amount of time. By contrast, convolutional neural networks are a type of deep neural network that uses convolutional, pooling, and fully connected layers to build the model architecture. It exhibits outstanding accuracy when trained on large datasets. However, when the quantity of training data is limited, the use of pre-trained networks for feature extraction may be helpful. Pre-trained models have been utilized in various medical imaging applications for image classification [31,32]. Figure 1 shows the concept of deep feature extraction using a pre-trained model (GoogLeNet).

Deep features are extracted by processing an image using a neural network and evaluating the activation of various layers. Classical machine-learning models can be used to categorize images using extracted deep features.

The following pre-trained networks were used in this study: DarkNet-19, DarkNet-53, DenseNet-201, EfficientNet-b0, GoogLeNet365, GoogLeNet, Inception-ResNet-v2, Inception-v3, MobileNet-v2, NASNet-Mobile, ResNet-101, ResNet-50, ResNet-18, ShuffleNet, SqueezeNet, and Xception. These models have distinctive qualities and capture various image characteristics, including local and global patterns. These models are useful for a variety of computer vision tasks, such as object detection, image segmentation, and image retrieval.

### 2.3. Optimal Feature Selection Using a Wrapper-Based Approach

Feature selection is crucial in many machine-learning applications because it directly affects model accuracy. Recognizing and using appropriate features that effectively define and categorize objects is critical. By contrast, incorporating irrelevant characteristics may diminish the accuracy. Consequently, determining the most valuable attributes and features is essential for improving the classification accuracy of the model.

The feature selection approach involves choosing an optimal subset of features from a larger collection to build the learning model. Then, using a specified criterion, the quality of the new subset is evaluated [33]. Many methodologies such as filter-based, wrapper-based, and embedding approaches can be used for feature selection [34]. These tactics not only improve classification accuracy, but they also reduce model complexity, leading to faster processing.

**Figure 2:**
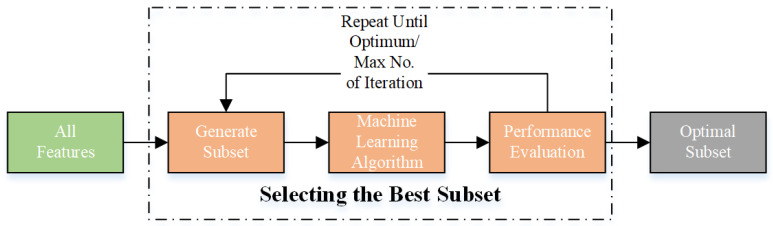
Workflow for the wrapper-based approach.

In this study, wrapper-based techniques were used to select the optimal feature subset to train the model. These algorithms are machine-learning techniques that evaluate the performance of a feature group when employed with a particular model, often known as the “wrapper” [35]. The algorithm evaluates the effect of the selected feature subset on the accuracy of the model. The algorithm selects the current feature subset or searches for a better subset based on the evaluation results. This approach is repeated until the best feature subset is obtained. Figure 2 illustrates the operation of the wrapper-based method. In this study, a range of metaheuristic algorithms and wrapper-based techniques were used to achieve optimum feature selection.

Metaheuristic methods attempt to estimate solutions for complicated issues. As they use several low-level heuristics to handle high-level optimization tasks, they are referred to as “meta” [36]. The MPA, GNDO, SMA, EO, MRFO, ASO, HHO, HGSO, PFA, and PRO are examples of metaheuristic algorithms. Learning algorithms assess the performance of the generated feature subsets. Metaheuristics were employed as search algorithms to identify new optimum subsets [37]. Equation (1) specifies the cost functions of all the optimization strategies.

(1) $$\min(M)=\phi (1-\mathrm{Accuracy})+\gamma \left(\frac{\mathrm{No}.\;\mathrm{of}\;\mathrm{selected}\;\mathrm{features}}{\mathrm{Total}\;\mathrm{no}.\;\mathrm{of}\;\mathrm{features}}\right),$$

where ϕ and γ are the coefficients of each criterion [38].

#### 2.3.1. Marine Predators Algorithm (MPA)

MPA is a nature-inspired optimization approach that follows natural rules that determine the optimum foraging tactics and encounter rates between predators and prey in marine habitats. MPA simulation is based on the hunting and foraging habits of marine predators, such as sharks and dolphins [39,40]. Predation, reproduction, migration, and exploration are the four fundamental phases of the MPA. It aggressively searches for and collects food sources during the predation phase. The transmission of genetic material to offspring occurs during the reproduction phase. The exploration step involves scouring the search space for new locations. Finally, predators relocate to other regions during the migratory period.

#### 2.3.2. Generalized Normal Distribution Optimization (GNDO)

GNDO is a method of parameter optimization or data fitting to a generalized normal distribution [41]. A statistical distribution, called the generalized normal distribution, expands the normal (Gaussian) distribution by adding form factors that enable more adaptable data modeling. Determining the values that best match the provided data, or optimizing a specific objective function, are steps for optimizing the parameters in a generalized normal distribution [42]. Many methods, including least-squares fitting and maximum likelihood estimation, can be used to complete the optimization process. The underlying distribution of the data can be learned, and statistical conclusions or forecasts can be drawn by optimizing the parameters of a generalized normal distribution. When data display non-normal features or require a more flexible distribution for modeling, this optimization approach is used in several disciplines, including finance, engineering, and data analysis.

#### 2.3.3. Slime Mold Algorithm (SMA)

SMA is a computational optimization approach developed after studying the behavior of slime molds [43]. Slime molds are single-celled creatures capable of self-organization and emergent behavior. This allows them to tackle challenging issues, such as determining the shortest route between food sources [44]. To resolve optimization issues, the SMA imitates the foraging behavior of slime molds. It begins with a population of digital particles that stand in for the individual slime molds. These particles travel across the search area while leaving a pheromone trail in their wake. Exploration, pattern generation, and exploitation are the three key phases of this algorithm [45]. The search space is randomly explored by particles during the exploration phase, leaving a pheromone trail in their wake. Pheromone trails attract particles during the pattern generation phase, prompting them to gather and create patterns. Finally, depending on the pheromone trails, the particles converge toward the most promising locations in the search space during exploitation. The SMA has several benefits, including versatility when solving continuous and discrete optimization issues, parallelizability, and resilience when addressing challenging and dynamic problem domains.

#### 2.3.4. Equilibrium Optimizer (EO)

EO is a metaheuristic optimization method based on the physical concept of equilibrium proposed by Faramarzi et al. [46,47]. The goal is to simulate the equilibrium state of a physical system to determine the optimum solution for a given issue. The optimization issue is represented as a population of individuals, each corresponding to a potential solution. Based on their fitness levels and the concept of equilibrium, the program iteratively adjusts the placement of these individuals. EO is divided into initialization, position update, and equilibrium update. The initial population of individuals is randomly generated during the startup process. The location of each individual is modified during the position–update step, depending on its present position, the positions of other individuals, and a series of mathematical equations derived from physical principles. This updating process attempts to explore the search space and converge on superior solutions. This method then modifies the parameters associated with equilibrium during the equilibrium update phase to achieve a balanced state in the population.

#### 2.3.5. Manta-Ray Foraging Optimization (MRFO)

MRFO is a metaheuristic optimization technique inspired by nature and the foraging behavior of manta rays. The manta ray is renowned for its effective foraging techniques [48]. The MRFO algorithm simulates the foraging behavior of manta rays to solve optimization issues. The potential solutions are represented as a set of rays, each corresponding to a candidate solution, using a population-based technique [49]. These beams scour the search area to find the best solution. Numerous crucial phases are present in the MRFO algorithm. First, a starting population of rays is randomly created within the search space. Each ray represents a potential resolution. The rays then travel across the search area using various foraging techniques motivated by manta-ray behavior. These tactics include searching for food sources, avoiding hazards, and preserving social contact. The rays adjust their movements during the optimization process, depending on their own experience and the collective wisdom of the population. They alter their placement and speed to investigate interesting areas and obtain better solutions. The algorithm uses a fitness evaluation technique to determine the quality of each ray’s position and directs the exploration and exploitation stages.

#### 2.3.6. Atom Search Optimization (ASO)

ASO is a newly developed physics-inspired metaheuristic optimization method designed to address a wide range of optimization issues [50]. It is motivated by fundamental molecular dynamics. The atomic motion model found in nature, wherein atoms interact through interaction forces originating from the Lennard–Jones potential, and constraint forces arising from the bond–length potential, is theoretically modeled and imitated by the ASO. The ASO is straightforward and simple to use. Further details on the algorithm can be found in [50,51].

#### 2.3.7. Harris Hawks Optimization (HHO)

Heidari et al. [52] proposed the HHO algorithm in 2019. It is a nature-inspired optimization algorithm based on the hunting behavior of the Harris’s hawk, a raptor species. A population-based optimization technique, the HHO algorithm, simulates the social structures and foraging habits of Harris’s hawks. In the wild, Harris’s hawks engage in intriguing group behaviors when hunting. They use a cooperative hunting technique in which certain hawks lead the group toward the prey, while others follow. The HHO simulates this behavior by categorizing the population into leaders and followers. Leaders seek potential solutions within the search space. Meanwhile, followers alter their positions based on the leaders’ information and they update their positions. This hierarchical structure allows for compelling search space exploration and exploitation. The HHO algorithm uses various operators to mimic the numerous hunting strategies employed by Harris’s hawks, including location updates, prey capture, and knowledge transfer. Using these operators iteratively optimizes a specified goal function. Numerous optimization issues, such as numerical optimization, engineering design, and feature selection, have been addressed using HHO [53].

#### 2.3.8. Henry Gas Solubility Optimization (HGSO)

HGSO is a revolutionary metaheuristic algorithm that solves difficult optimization problems by mimicking the behavior regulated by Henry’s law [54]. Henry’s law is a fundamental gas law that describes the amount of a given gas dissolved in a particular type and volume of liquid at a given temperature. To balance exploitation and exploration in the search space and prevent local optima, the HGSO algorithm mimics the huddling behavior of gas. Further details regarding HGSO can be found in [54].

#### 2.3.9. Path Finder Algorithm (PFA)

PFA is a metaheuristic optimization technique developed to address various optimization issues. The PFA is inspired by the collective movement of animals and it replicates the leadership structure of swarming to determine the best possible feeding location, or it locates prey [55]. The PFA uses multiple processes such as pheromone deposition, probabilistic selection, and local search algorithms to improve its exploration and exploitation capabilities. It also has adaptive settings and self-adjusting technologies that dynamically govern the search process. Further details regarding PFA can be found in [55].

#### 2.3.10. Poor and Rich Optimization (PRO)

The interactions between the wealthy and the poor, as they work to increase their wealth and economic standing, serve as the basis for the PRO algorithm [56]. The rich always try to expand the wealth gap by gaining more wealth through various means. By contrast, the poor want to increase their money and narrow the wealth gap by imitating the affluent. It is vital to emphasize that this conflict is continuous and that people from both groups have the ability to move between rich and poor categories. The algorithm uses a two-stage approach. During the exploration phase, rich and poor people explore the search area individually. To improve their seeking capacity, the poor mimic the movements and actions of the wealthy. Using a cooperative search approach, the exploitation phase focuses on exploitation. Rich people impart their expertise and information to help poor people increase their search efficiency. Further details regarding PRO can be found in [56].

## 3. Proposed Framework for Breast Cancer Detection

**Figure 3:**
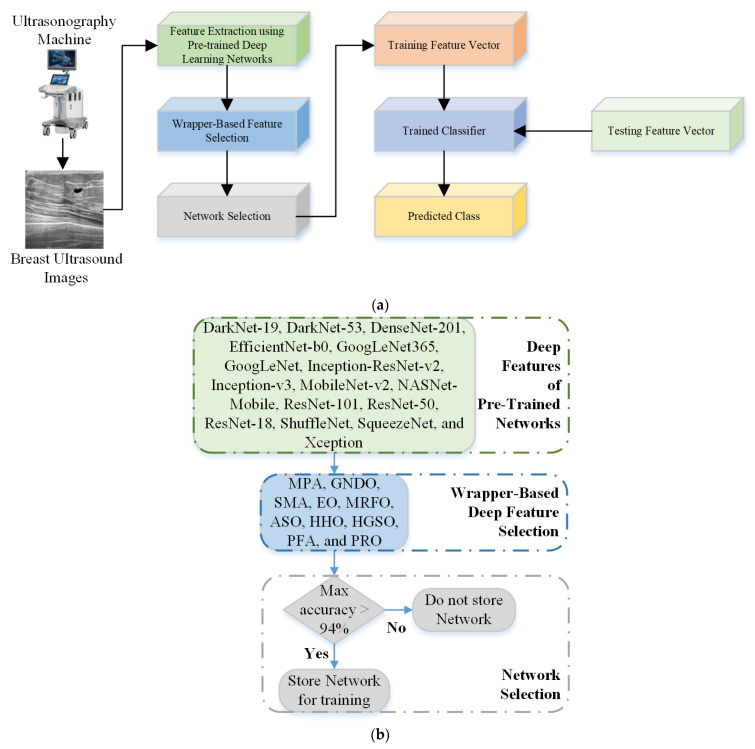
(**a**) Proposed flowchart for the diagnosis of BC; (**b**) Network selection approach.

BU is a reliable tool that is often used as a diagnostic technique following an abnormal mammogram or clinical breast examination. It provides additional information to guide subsequent investigations such as a fine-needle aspiration, core biopsy, or surgical excision. This study aims to develop an intelligent machine-learning model capable of detecting BC and classifying it into further classes (benign and malignant). After acquiring the images from the machine, pre-trained models were used to extract the deep features, as discussed in Section 2.2. Next, ten wrapper-based optimization algorithms, MPA, GNDO, SMA, EO, MRFO, ASO, HHO, HGSO, PFA, and PRO, were used to retrieve the most informative features, as discussed in Section 2.3. In the next step, a network selection algorithm with a classification accuracy above 94% was employed to select and concatenate the deep features. A complete flowchart of the proposed framework is presented in Figure 3.

An optimization algorithm with an accuracy of more than 94% for the deep features of a single network was used to select deep-learning networks during the network selection phase. Furthermore, the classical machine-learning SVM model was utilized for BC classification [57,58].

## 4. Results and Discussion

In this study, the proposed wrapper-based deep network selection method was employed to improve the performance of BU images for detecting and distinguishing BC types. An available online BC dataset was used to test the proposed framework [30], as described in Section 2.1. As previously discussed, discrimination between the BU images of BC subtypes was achieved by employing the deep features of multiple pre-trained models. The wrapper-based algorithms outlined above (MPA, GNDO, SMA, EO, MRFO, ASO, HHO, HGSO, PFA, and PRO) were used to retrieve important features. MATLAB 2023a, running on a computer with the following specifications, was used for all processing and analyses, and it comprised the following: 32 GB of RAM, 1 TB SSD, 11th Generation, Intel(R) Core (TM) i7-10700, and 64-bit Windows 11 Pro. The 0.2 holdout validation approach was used to divide the dataset into training and testing datasets. To avoid overfitting, 20% of data were not used in training. The population size of each algorithm was set to 10; 100 was the maximum number of iterations. The values of ϕ and γ were set as 0.99 and 0.01, respectively [38]. All other parameters of each optimization algorithm are listed in Table 2.

**Figure 4:**
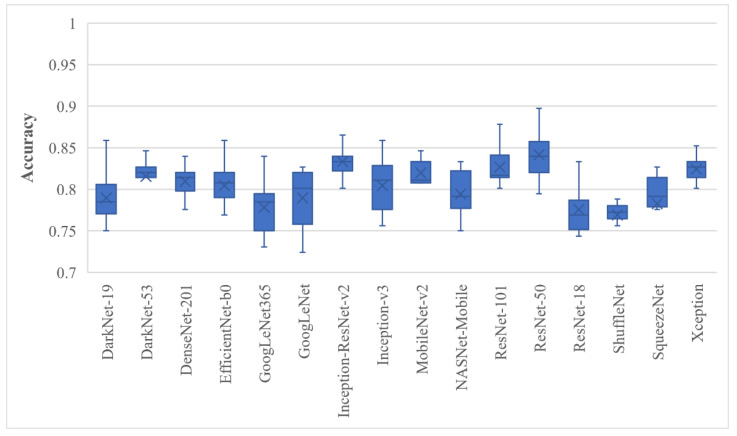
BU image classification that used the full deep features of various pre-trained models trained with SVM; whiskers represent the range.

First, the deep features of all 16 models were extracted before the softmax layer and they were used for training the SVM model; the results are presented in Figure 4.

**Figure 5:**
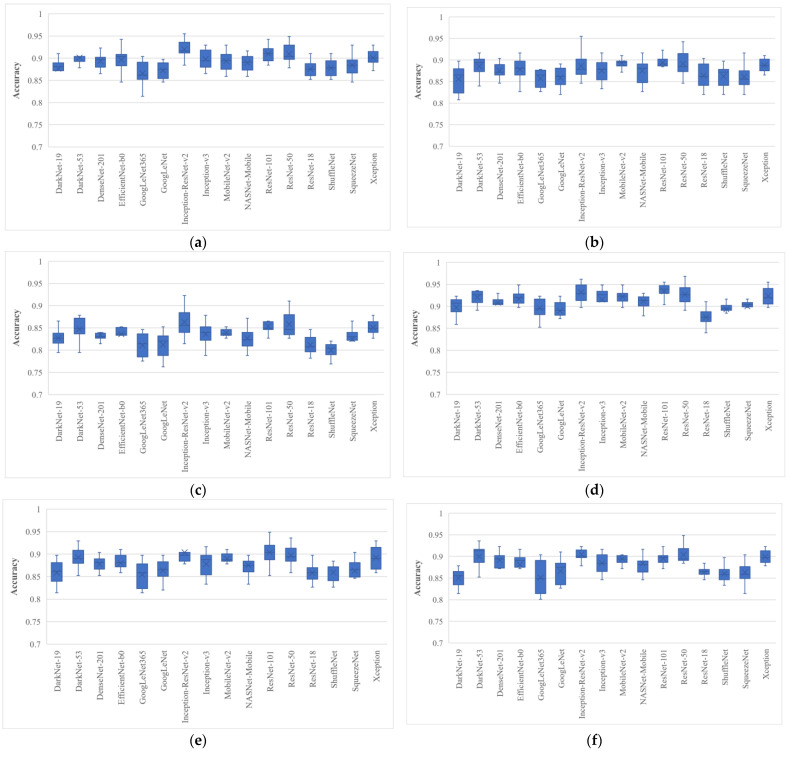

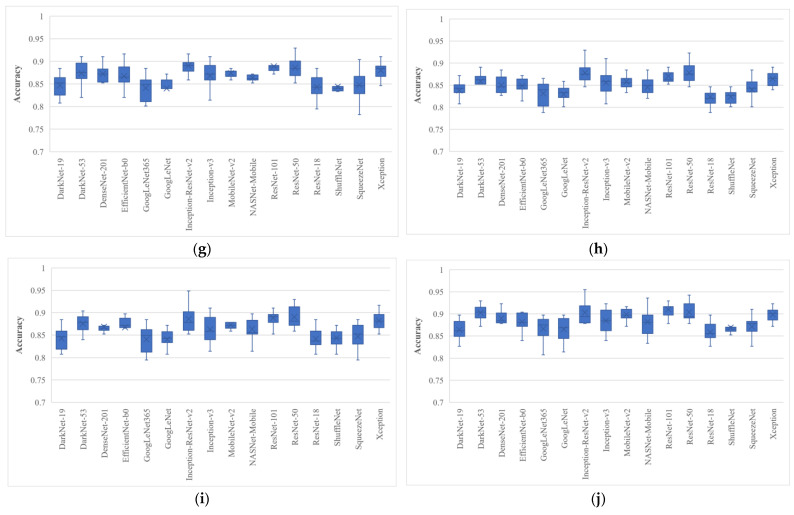
Classification accuracy of various wrapper-based optimization approaches for BU images: (**a**) MPA; (**b**) GNDO; (**c**) SMA; (**d**) EO; (**e**)MRFO; (**f**) ASO; (**g**) HHO; (**h**) HGSO; (**i**) PFA; (**j**) PRO, where whiskers represent the range.

Figure 4 shows box plots of the classification accuracy for the full features of the pre-trained models. The BU dataset was randomly divided ten times in the 80% to 20% ratio; 20% of the data were retained and not used for model training. The results depicted in Figure 4 concluded that the ResNet-50 and Inception-ResNet-v2 trained SVMs achieved the highest average classification accuracy of 84.17 ± 3.08% and 83.33 ± 2.79%, respectively. The proposed approach was then employed, and the results are shown in Figure 5.

After analyzing the results, it was found that the EO algorithm produced the highest classification accuracy of 96.79%, trained with only 562 features, and had a minimum average classification accuracy of 89.1% when trained with a feature vector with a size of 570 with ResNet-50 deep features. Similarly, the Inception-ResNet-v2 had the second highest classification accuracy of 96.15% for a single run. It is also clear from Figure 5 that all the pre-trained models achieved their best accuracies with the EO algorithm. The average classification accuracy and average feature number of each pre-trained model for each optimization algorithm are listed in Table 3 and Table 4, respectively.

**Figure 6:**
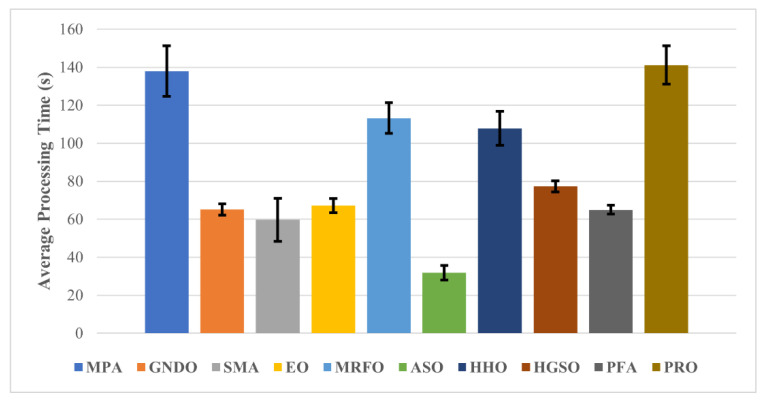
Average processing time of each wrapper-based method.

The average processing times of all algorithms for the pre-trained deep-learning model feature optimization are shown in Figure 6.

After carefully analyzing the outcomes of processing time, it can be observed that the ASO uses the least computation time of only 31.79 ± 3.78 s, with a reasonable average classification accuracy of more than 88%. By contrast, the best optimization algorithm (EO) used less than 70 s to process the data, with an average accuracy of 94.69 ± 2.48, for ResNet-50 deep features. A comparison of the proposed approach with other BC detection approaches is presented in Table 5.

BU imaging is critical for evaluating breast lesions in the presence of palpable lumps or breast discomfort. Mammography is widely considered the primary method for the early diagnosis of BC. However, its usefulness in young women with dense breast tissue is limited. Furthermore, BU is an ideal diagnostic approach for minimizing the hazards of mammography radiation exposure [64].

Furthermore, BU imaging offers comprehensive information on solid lesions. Cysts are the most common type of benign breast lesions in women. Anechoic, thin-walled, and well-circumscribed lesions are visible on BU images. In addition to being noninvasive, nonradioactive, and cost-effective, BU imaging is well tolerated by female patients. Deep-learning approaches have advanced to the point where they can help in BC diagnosis [65,66,67]. In one study [63], the authors used the deep features of a pre-trained model, and the filter-based method, minimum-redundancy maximum-relevance, was used to extract the optimal feature subset; the model achieved an accuracy of 95.6% for an augmented dataset. Similarly, Alduraibi [29] applied the ReliefF filter-based method to determine the relevant features of a pre-trained model and achieved accuracies of 94.57 and 90.39% for augmented and original datasets, respectively. Regarding filter-based approaches, the optimal subset is selected based on its relevance to the dependent variable; the machine-learning algorithm is not used to select the features. Therefore, a favorable outcome cannot be guaranteed. However, in the case of wrapper-based methods, extracting the optimal features by testing them using a machine-learning model guarantees reliability and high classification performance. A comparison of the proposed study with previously published works showed the superiority of the proposed approach in terms of a high classification rate (a rise of 1.05% compared with the study that had the highest classification accuracy [61]). Therefore, the proposed BU framework may help practitioners to detect BC quickly and effectively.

## 5. Conclusions

In this study, a wrapper-based BU image classification methodology was designed to improve the BC detection capability in women. The deep features of the 16 pre-trained models were extracted, and ten optimization algorithms (MPA, GNDO, SMA, EO, MRFO, ASO, HHO, HGSO, PFA, and PRO) were used to retrieve the optimal features using the SVM classifier. The classification performance of each pre-trained model was significantly improved, and the size of the feature vector was decreased using optimization algorithms. A network selection strategy was used to determine the best features of the pre-trained network. After comprehensive testing and analysis, the EO algorithm produced the highest classification rate for each pre-trained model. It produced the highest classification accuracy of 96.79%, trained with a deep feature vector, with a size of 562 in the ResNet-50 model. Similarly, the Inception-ResNet-v2 had the second highest classification accuracy of 96.15% for a single run using the EO algorithm. Therefore, the proposed BU framework may be helpful for automatic BC identification.

## Figures and Tables

**Table 1:** Information on the online BU dataset [30].

	Benign	Malignant	Normal
BU images	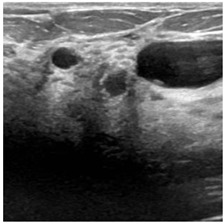	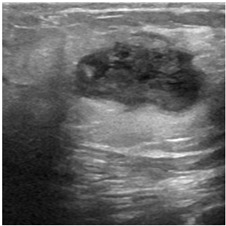	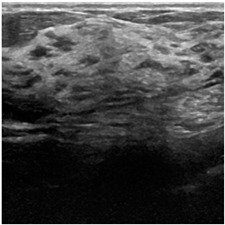
Number of images per class	437	210	133

**Table 2:** MPA, GNDO, SMA, EO, MRFO, ASO, HHO, HGSO, PFA, and PRO parameters.

MPA	GNDO	SMA	EO	MRFO	ASO	HHO	HGSO	PFA	PRO
Fish aggregating devices effect = 0.2	Lower bound = 0	Lower bound = 0	Constant 1 = 2	Somersault factor = 2	Depth weight = 50	Lower bound = 0	Number of gas types = 2	Lower bound = 0	Lower bound = 0
Constant = 0.5	Upper bound = 1	Upper bound = 1	Constant 2 = 1		Multiplier weight = 0.2	Upper bound = 1	Constant = 1	Upper bound = 1	Upper bound = 1

**Table 3:** Classification accuracy of various wrapper-based optimization algorithms with deep features of various pre-trained deep-learning models (data represented as the mean ± the standard deviation of ten runs).

Deep-Learning Network	MPA	GNDO	SMA	EO	MRFO	ASO	HHO	HGSO	PFA	PRO	Full Features
DarkNet-19	87.6 ± 2.8	85.6 ± 3.3	82.7 ± 2.1	89.7 ± 2.7	85.9 ± 3	85 ± 2.2	84.7 ± 2.6	83.8 ± 2.4	84.2 ± 2.5	86.4 ± 2.4	79 ± 3.2
DarkNet-53	90.1 ± 1.7	88.5 ± 2.6	84.7 ± 2.8	92.1 ± 1.7	89.3 ± 2.5	90 ± 3.2	87.4 ± 2.8	85.8 ± 2.3	87.6 ± 2	90.3 ± 1.8	81.5 ± 2.7
DenseNet-201	89.4 ± 1.7	87.6 ± 1.8	83.5 ± 1.8	90.8 ± 1.9	87.8 ± 1.8	89.2 ± 1.8	87.2 ± 1.9	85 ± 2.1	86.9 ± 1.3	89 ± 2.4	81 ± 2.2
EfficientNet-b0	89.7 ± 2.9	87.8 ± 3.1	83.6 ± 3.3	91.7 ± 2.4	88 ± 2.5	88.1 ± 3	86.7 ± 3	84.9 ± 2.9	86.7 ± 3	88.2 ± 3.6	80.4 ± 3.5
GoogLeNet365	86.6 ± 2.8	85.7 ± 2.2	81.1 ± 2.7	89.6 ± 2.3	85.4 ± 3.1	85.1 ± 4.1	84.1 ± 3	83.1 ± 2.9	84 ± 3.1	86.7 ± 2.9	77.8 ± 3.5
GoogLeNet	87.2 ± 2	85.9 ± 2.6	81.2 ± 3	89 ± 2.6	86.4 ± 2.6	86.7 ± 3.1	84 ± 2.6	82.9 ± 2.4	84 ± 2.7	86.5 ± 2.7	79 ± 3.7
Inception-ResNet-v2	92 ± 2.2	88.7 ± 3.2	86.3 ± 3.4	94.2 ± 2.3	90.4 ± 2.7	90.5 ± 2.1	89 ± 2.2	87.8 ± 2.4	88.6 ± 3.1	90.3 ± 2.8	83.3 ± 2.8
Inception-v3	89.8 ± 2.3	87.6 ± 2.8	83.6 ± 2.9	91.5 ± 2.9	87.8 ± 2.8	88.5 ± 2.4	86.8 ± 3.3	85.6 ± 3	86.3 ± 3.2	88.5 ± 2.9	80.4 ± 3.4
MobileNet-v2	89.4 ± 2.2	89.3 ± 1.5	83.8 ± 1.8	92.2 ± 1.5	89 ± 1.6	89.5 ± 2.3	87.4 ± 1.4	85.8 ± 1.7	87.3 ± 1.3	89.9 ± 1.4	82 ± 1.5
NASNet-Mobile	89 ± 1.9	87.5 ± 3.1	82.7 ± 2.6	91.2 ± 2.2	87.6 ± 2.4	88.4 ± 2.8	86.4 ± 2.4	84.6 ± 2.8	86.3 ± 2.6	88.2 ± 3.4	79.4 ± 3.1
ResNet-101	91 ± 1.8	89.4 ± 2	85.4 ± 1.8	93.4 ± 2	90.4 ± 2.7	89.6 ± 1.4	88.9 ± 2.5	87.1 ± 1.9	88.8 ± 2	91 ± 2.1	82.7 ± 2.5
ResNet-50	91 ± 2.5	89.2 ± 2.9	85.8 ± 3	94.7 ± 2.5	89.7 ± 2.3	90.4 ± 2.1	88.6 ± 2.3	87.8 ± 2.4	89 ± 2.6	90.4 ± 2.2	84.2 ± 3.1
ResNet-18	87.6 ± 1.9	86.4 ± 2.8	81.2 ± 2.2	87.5 ± 2.1	85.8 ± 2.1	86.4 ± 1.7	84.4 ± 2.9	82.4 ± 2.3	84.2 ± 2.5	85.8 ± 2.3	77.6 ± 3
ShuffleNet	87.8 ± 2	86.2 ± 2.7	80 ± 1.6	89.4 ± 1.7	86 ± 2.1	86 ± 2.2	84.4 ± 2.6	82.4 ± 1.5	84.4 ± 2.1	86.9 ± 1.7	76.9 ± 1.7
SqueezeNet	88.5 ± 2.6	86.2 ± 2.8	82.5 ± 2.9	90 ± 2.1	86.3 ± 3	86.3 ± 2.8	84.7 ± 3.6	84 ± 3.1	84.7 ± 3.3	87.1 ± 2.6	78.3 ± 4.6
Xception	90.1 ± 1.8	88.8 ± 1.6	85.1 ± 1.7	92.4 ± 2.3	89.2 ± 2.6	89.9 ± 1.6	88 ± 2	86.5 ± 1.7	88.2 ± 2.1	89.9 ± 1.8	82.4 ± 1.5

**Table 4:** Feature vector sizes of various wrapper-based optimization algorithms for deep features of various pre-trained deep-learning model (data represented as the mean ± the standard deviation of ten runs).

Deep-Learning Network	MPA	GNDO	SMA	EO	MRFO	ASO	HHO	HGSO	PFA	PRO	Full Features
DarkNet-19	309 ± 128	481 ± 14	386 ± 172	257 ± 49	368 ± 155	489 ± 18	308 ± 95	192 ± 124	487 ± 21	341 ± 156	1000 ± 0
DarkNet-53	425 ± 161	500 ± 15	374 ± 212	299 ± 57	491 ± 91	506 ± 15	502 ± 91	490 ± 158	498 ± 18	492 ± 29	1024 ± 0
DenseNet-201	783 ± 245	939 ± 21	790 ± 342	636 ± 93	1055 ± 231	954 ± 20	1071 ± 194	1033 ± 173	955 ± 25	948 ± 26	1920 ± 0
EfficientNet-b0	429 ± 130	612 ± 22	540 ± 222	391 ± 51	510 ± 163	637 ± 21	630 ± 150	500 ± 231	634 ± 22	618 ± 124	1280 ± 0
GoogLeNet365	321 ± 135	490 ± 14	294 ± 222	240 ± 53	498 ± 153	506 ± 15	469 ± 244	244 ± 150	501 ± 13	401 ± 139	1024 ± 0
GoogLeNet	386 ± 122	505 ± 17	337 ± 234	319 ± 49	523 ± 112	505 ± 16	481 ± 156	468 ± 153	498 ± 21	396 ± 134	1024 ± 0
Inception-ResNet-v2	405 ± 137	736 ± 19	500 ± 350	376 ± 62	567 ± 169	746 ± 20	529 ± 193	495 ± 203	753 ± 24	555 ± 216	1536 ± 0
Inception-v3	462 ± 132	990 ± 20	587 ± 455	540 ± 173	831 ± 189	1011 ± 26	771 ± 352	659 ± 345	1014 ± 28	735 ± 278	2048 ± 0
MobileNet-v2	533 ± 119	622 ± 18	527 ± 232	423 ± 59	633 ± 93	628 ± 15	650 ± 116	549 ± 237	634 ± 21	620 ± 16	1280 ± 0
NASNet-Mobile	357 ± 134	521 ± 10	361 ± 191	305 ± 75	511 ± 125	517 ± 11	396 ± 113	359 ± 206	522 ± 15	392 ± 184	1056 ± 0
ResNet-101	698 ± 261	996 ± 16	770 ± 428	573 ± 123	990 ± 360	1019 ± 18	913 ± 176	774 ± 303	1006 ± 28	970 ± 199	2048 ± 0
ResNet-50	737 ± 243	993 ± 30	789 ± 391	567 ± 46	968 ± 215	1005 ± 15	755 ± 286	857 ± 305	1014 ± 26	1016 ± 40	2048 ± 0
ResNet-18	202 ± 70	251 ± 11	256 ± 6	153 ± 20	266 ± 50	254 ± 11	244 ± 47	207 ± 69	253 ± 9	250 ± 11	512 ± 0
ShuffleNet	171 ± 58	260 ± 16	173 ± 106	140 ± 31	278 ± 74	269 ± 13	259 ± 54	227 ± 80	269 ± 12	242 ± 67	544 ± 0
SqueezeNet	229 ± 90	477 ± 10	190 ± 215	249 ± 38	388 ± 97	490 ± 13	348 ± 156	322 ± 218	480 ± 13	360 ± 186	1000 ± 0
Xception	652 ± 186	999 ± 26	938 ± 227	584 ± 49	793 ± 142	999 ± 30	826 ± 119	843 ± 322	1022 ± 25	992 ± 38	2048 ± 0

**Table 5:** Comparison of the proposed approach with other BC detection approaches.

Reference	Classes	Accuracy (%)
Liao et al. [59]	Benign and Malignant	92.95
Huang et al. [60]	Benign and Malignant	94.20
Yu et al. [61]	Benign, Malignant, Normal	95.74
Moon et al. [62]	Benign, Malignant, Normal	94.62
Alduraibi [29]	Benign, Malignant, Normal	94.57
Eroğlu et al. [63]	Benign, Malignant, Normal	95.6
This study	Benign, Malignant, Normal	96.79

## Data Availability

The data used to support the findings of this study are included in the article.
